# FSL‐MRS: An end‐to‐end spectroscopy analysis package

**DOI:** 10.1002/mrm.28630

**Published:** 2020-12-06

**Authors:** William T. Clarke, Charlotte J. Stagg, Saad Jbabdi

**Affiliations:** ^1^ Wellcome Centre for Integrative Neuroimaging, FMRIB, Nuffield Department of Clinical Neurosciences University of Oxford Oxford United Kingdom; ^2^ MRC Brain Network Dynamics Unit University of Oxford Oxford United Kingdom

**Keywords:** Bayesian fitting, MRS, MRSI, open‐source, spectroscopy

## Abstract

**Purpose:**

We introduce FSL‐MRS, an end‐to‐end, modular, open‐source MRS analysis toolbox. It provides spectroscopic data conversion, preprocessing, spectral simulation, fitting, quantitation, and visualization.

**Methods:**

The FSL‐MRS package is modular. Its programs operate on data in a standard format (Neuroimaging Informatics Technology Initiative [NIfTI]) capable of storing single‐voxel and multivoxel spectroscopy, including spatial orientation information. The FSL‐MRS toolbox includes tools for preprocessing of raw spectroscopy data, including coil combination, frequency and phase alignment, and filtering. A density matrix simulation program is supplied for generation of basis spectra from simple text‐based descriptions of pulse sequences. Fitting is based on linear combination of basis spectra and implements Markov chain Monte Carlo optimization for the estimation of the full posterior distribution of metabolite concentrations. Validation of the fitting is carried out on independently created simulated data, phantom data, and three in vivo human data sets (257 single‐voxel spectroscopy and 8 MRSI data sets) at 3 T and 7 T. Interactive HTML reports are automatically generated by processing and fitting stages of the toolbox. The FSL‐MRS package can be used on the command line or interactively in the Python language.

**Results:**

Validation of the fitting shows low error in simulation (median error of 11.9%) and in phantom (3.4%). Average correlation between a third‐party toolbox (LCModel) and FSL‐MRS was high (0.53‐0.81) in all three in vivo data sets.

**Conclusion:**

The FSL‐MRS toolbox is designed to be flexible and extensible to new forms of spectroscopic acquisitions. Custom fitting models can be specified within the framework for dynamic or multivoxel spectroscopy. It is available as part of the FMRIB Software Library.

## 1 INTRODUCTION

Recent years have seen the emergence and rapid progress of new MRS technologies, including spectral editing,^1^ MRS imaging,^2^, ^3^ time‐resolved functional MRS,^4^ diffusion‐weighted MRS,^5^ and MRS fingerprinting.^6^ Magnetic resonance spectroscopy is, therefore, starting to have a range of techniques comparable to those of conventional proton MRI, but with the added benefit of being able to quantify specific chemical compounds. However, unlike modern MRI‐based neuroimaging, MRS lacks standard data formats (eg, Neuroimaging Informatics Technology Initiative [NIfTI]), as well as standard preprocessing and analysis pipelines suitable for use by nonexpert users (eg, FSL,^8^ SPM,^9^ or AFNI^10^). This restricts the use of MRS in research, particularly in neuroscience, by requiring expertise in MRS acquisition, data analysis, and computing. Current processing toolboxes are typically linear and linearly dependent, lacking modularity or a standardized data format. It is therefore difficult to customize processing pipelines, inspect the results of single steps of a pipeline, or combine steps from different toolsets.

The tools currently available and commonly in use for processing, fitting, and visualization of spectra (eg, Refs. 11, 12) suffer from one or more of several limitations, namely:

- They may be black‐box, closed‐source implementations, sometimes with monetary cost;
- They may require licensed software to run, which is not universally deployable;
- They often require high user interaction, either through a graphical user interface or the need for setting and understanding many options;
- They have fixed forward‐fitting models. Modifications require MRS and computing expertise; and/or
- They have limited or no handling of MRSI data, with no parallelization available.

For these reasons, currently available software is not easily extensible to new forms of MRS, such as high‐resolution, high‐voxel‐count MRSI, or time‐series modeling of functional MRS or diffusion‐weighted MRS.

In this work we present a new Python‐based MRS fitting and processing tool, FSL‐MRS. The toolbox is open‐source, free as part of the FSL software package,^8^ and operates with a scriptable command line or interactive interface. It implements a modular approach to spectroscopy analysis with a common data format, allowing integration with other neuroimaging tools. Steps are parallelizable for MRSI data. The FSL‐MRS package is end‐to‐end, comprised of modules for data conversion, preprocessing, basis spectra simulation, fitting, quantification, and visualization.

The FSL‐MRS fitting module works on the principle of linear combination of precalculated basis spectra.^13^ In keeping with FSL’s tradition of favoring Bayesian inference approaches,^17^ our tool calculates full posterior distributions of the fitted metabolite concentrations using a Markov chain Monte Carlo (MCMC) algorithm, specifically Metropolis‐Hastings.^18^ The full posterior distributions can be used in further analysis, allowing efficient propagation of fitting uncertainties into downstream modeling and statistical analyses. Parameter covariances are also available from the fitting output, and point estimates of concentration and uncertainties may be calculated using appropriate summary statistics. The FSL‐MRS toolbox incorporates an interactive reporting interface that uses modern data‐science visualization tools.

In this work we describe the FSL‐MRS components, interface and output, and describe the fitting model and approach. A validation of the tool’s fitting estimates is carried out on widely available simulated data, in phantom, and on three in vivo data sets at 3 T and 7 T, spanning 265 subjects.

## 2 METHODS

### 2.1 Data conversion and format

The FSL‐MRS toolbox operates on a modular processing principle. Modularity allows custom third‐party additions to the processing pipeline without the need to alter the FSL‐MRS package or adhere to FSL‐MRS‐imposed code conventions, languages, or possible limitations.

To enable this workflow, FSL‐MRS processing and fitting operates on MRS data stored in the NIfTI format.^7^ The NIfTI format permits the storage of data resolved into three spatial dimensions, in addition to a time dimension and two further unspecified dimensions. The MRS and MRSI time‐domain data may therefore be stored using the format (and will allow analysis of functional MRS and diffusion‐weighted MRS data in the future). Data are loaded from, and written to, file after each operation. Additional required meta‐data are stored in, read from, and written to JavaScript object notation “sidecar” files, as specified by the Brain Imaging Data Structure format.^19^

The FSL‐MRS package provides the spec2nii program to convert from existing data formats to NIfTI. Spec2nii currently supports seven formats specified in Supporting Information Table S1. Spectroscopy volume position information is translated into the NIfTI “qform” field, where it is available in the original format.

### 2.2 Modular end‐to‐end processing

**FIGURE 1:**
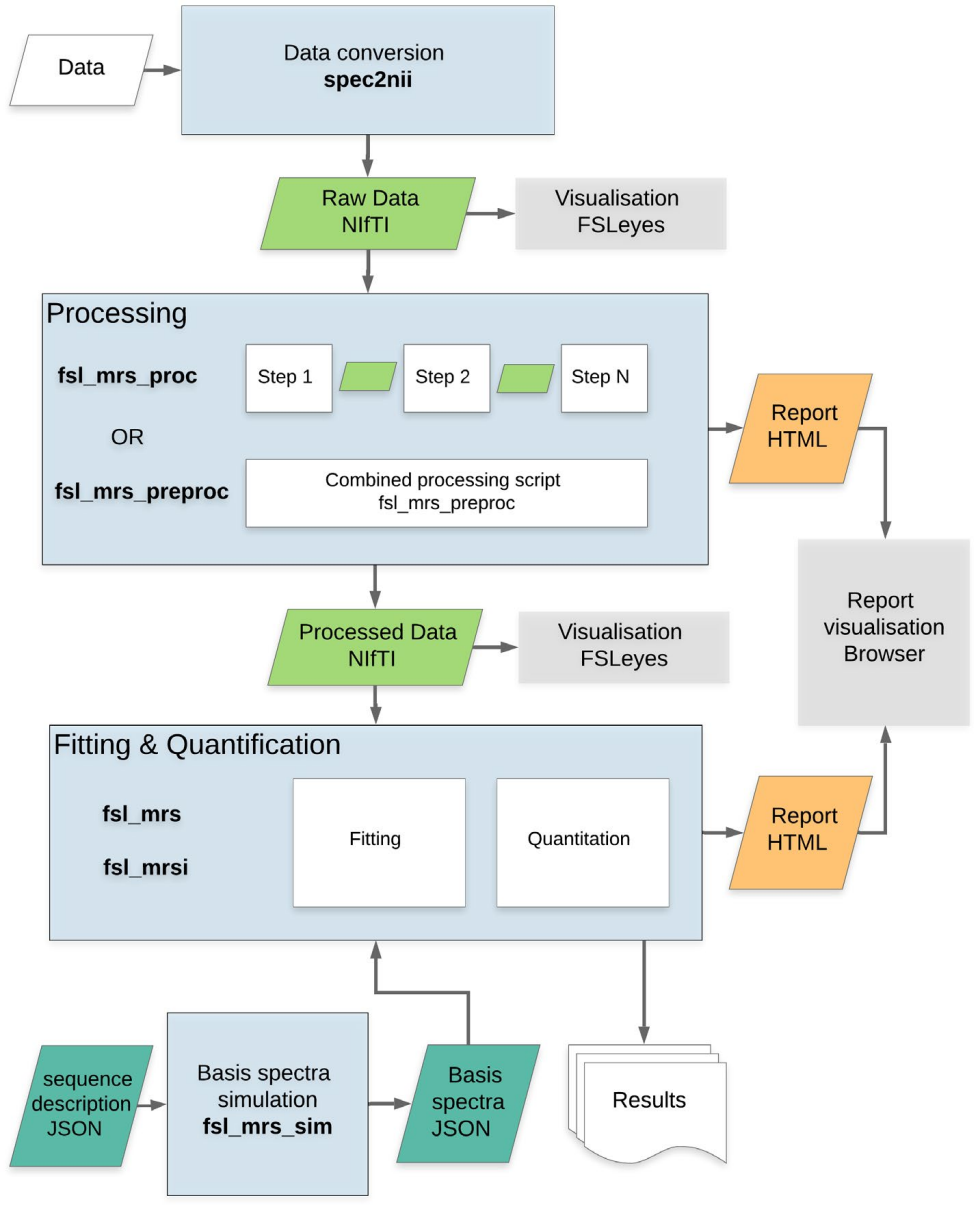
The FSL‐MRS organization and workflow. Raw data in proprietary or other formats are converted to NIfTI (Neuroimaging Informatics Technology Initiative) by spec2nii. Processing can then be carried out in stages, operating on NIfTI files using fsl_mrs_proc, or in a single Python script fsl_mrs_preproc for standard single‐voxel spectroscopy (SVS) sequences. Basis spectra can be generated for fitting using fsl_mrs_sim, given a JavaScript object notation (JSON) description for the sequence. Fitting and quantitation are then carried out by fsl_mrs and fsl_mrsi as appropriate. Interactive HTML reports are generated for viewing in the user’s browser. Spectroscopy data in NIfTI format can be viewed overlaid with other MR contrasts in FSLeyes

The FSL‐MRS toolbox provides a complete set of command line tools for spectroscopy analysis. Here we define *processing* as the steps required to make single‐voxel spectroscopy (SVS) or reconstructed MRSI k‐space data ready for *fitting*. *Basis spectra creation* is the process of using quantum mechanical simulations (or other methods) to create numerical descriptions of a metabolite’s response to a specific MRS pulse sequence. *Fitting* is the process of estimating relative metabolite concentrations from the processed spectrum and the basis spectra. *Quantification* turns those relative concentrations into real‐world interpretable units of concentration. *Display* incorporates viewing of the data and results at all stages of the process. Figure 1 shows an overview of the tool’s workflow.

#### 2.2.1 Processing

The FSL‐MRS package provides tools for all of the processing operations recommended in the community‐driven consensus paper (Tables 2‐4 of Near et al^20^). These tools are accessed through the command line by *fsl_mrs_proc* and are found in Table 1. Coil combination is performed through the whitened singular value decomposition algorithm,^21^, ^22^ spectral alignment by spectral registration,^23^ and nuisance peak removal by Hankel Lanczos singular value decomposition (HLSVDPRO).^24^ *fsl_mrs_proc* operations are applied sequentially to data stored in NIfTI format. Operations can be combined, in order, to form a repeatable batch processing script.

**TABLE 1:** Processing operations available using the fsl_mrs_proc command line tool

fsl_mrs_proc operation	Description	References
coilcombine	Combine individual coils of receiver phased array	Refs. 21, 22
average	Average FIDs, with optional complex weighting	
align	Phase and frequency‐align FIDs using spectral registration	Ref. 23
align‐diff	Phase and frequency‐align subspectra based on addition or subtraction of subspectra (eg, for ISIS localization)	Ref. 23
ecc	Eddy current correction using a water phase reference scan	Ref. 25
remove	Remove peak (typically residual water) using HLSVD	Ref. 24
tshift	Shift/resample in time domain	
truncate	Truncate/pad time‐domain data by an integer number of points	
apodize	Apply choice of apodization function to the data	
fshift	Frequency domain shift	
unlike	Identify outlier FIDs and remove based on similarity metric	Ref. 14
phase	Zero‐order phase spectrum by phase of maximum point in range	
subtract	Subtract two FIDs	
add	Add two FIDs	

Abbreviations: HLSVD, Hankel Lanczos singular value decomposition; ISIS, image‐selected in vivo spectroscopy.

In addition to the flexibility offered by this script, FSL‐MRS also provides a prepackaged processing pipeline for nonedited single‐voxel data (*fsl_mrs_preproc*), which runs all appropriate steps with one command‐line operation.

#### 2.2.2 Basis spectra simulation

Fitting in FSL‐MRS works on the principle of linear combination (LC) modeling (see section 2.4), which requires that the user provide the algorithm with simulated (or measured) numerical responses of metabolite spin systems to the MRS pulse sequence being used. These responses are specific to the pulse sequence, the sequence timings, and the sequence RF pulse envelopes, and are known as basis spectra. Basis spectra must preserve the relative signal amplitude between metabolites.

The FSL‐MRS package provides an interface (*fsl_mrs_sim*) for the creation of basis spectra when provided with a description of the sequence timings, RF pulses, slice‐selection gradients, and rephasing gradient areas. The RF pulses may have arbitrary amplitude and phase modulation (ie, be nonideal). The description is provided in a JavaScript object notation format; examples are provided in the software documentation. The simulation is based on the extended one‐dimensional projection implementation of density matrix simulations.^26^, ^27^ Unwanted coherences are removed with a coherence order filter.^28^ Standard literature values for common spin‐system chemical shifts and coupling constants are included in the software.^29^, ^30^

The *fsl_mrs_sim* script outputs a JavaScript object notation file for each simulated metabolite, which may be loaded by FSL‐MRS’s fitting modules. The FSL‐MRS toolbox also accepts LCModel (.BASIS) and jMRUI (.txt) basis spectra formats.

#### 2.2.3 Fitting and quantification

Fitting in FSL‐MRS is provided by two command‐line interfaces: *fsl_mrs* (for SVS data) and *fsl_mrsi* (for MRSI data). Additional interfaces will be added in the future for other types of MRS (eg, diffusion‐weighted MRS, functional MRS). Fitting is carried out on each voxel of data independently. The user may optionally specify the limits of fitting (in ppm), the order of the complex polynomial baseline (see standard fitting model), whether to add default macromolecular peaks (at 0.9, 1.2, 1.4, 1.7, 2.08, and 3.0 ppm), the optimization algorithm (see section 2.4.2). Metabolites in the basis spectra file may be optionally excluded by the user and, for output purposes only, metabolites may be combined.

For meaningful quantification, the user must supply a processed, unsuppressed, water data set, and for transverse relaxation–compensated concentrations, the user must supply the sequence TE and tissue volume fractions.^31^, ^32^ Water signal amplitude (*S*_H2O_obs_ in Refs 20, 31, 32) is calculated using numerical integration of the real part of the phase‐corrected and eddy current–corrected, unsuppressed water spectrum. Water‐scaled concentrations are calculated by taking the ratio of the integrated signal of a scaled reference metabolite basis spectrum (*S*_M_obs_ in Refs 20, 31, 32; defaults to creatine between 2 and 5 ppm). The scripts *svs_segment* and *mrsi_segment* can calculate tissue‐volume fractions within each voxel given an appropriate T_1_‐weighted structural image. Default values for water concentration, tissue–water densities, and water and metabolite T_2_ time constants are provided for 3T and 7T field strengths. Hardcoded constants, correct as of time of publication, may be found in Supporting Information Tables S2‐S4), and the values that are correct for the current version may always be found in the source‐code module *fsl_mrs.utils.constants* or as part of the online documentation. These defaults may be overridden in the interactive or python interface. Concentrations can be expressed as a ratio to an arbitrary internal reference metabolite (or combination of metabolites) or in molar (mol/dm^3^) or molal (mol/kg) units.

The FSL‐MRS fitting outputs the SNR and linewidths (FWHM) for each fitted metabolite. The SNR ratio is calculated as the ratio of the peak height of the fitted metabolite basis spectrum over the SD of a pure noise region of the spectrum after a matched filter has been applied to both.^33^ The matched filter and linewidth are calculated for each metabolite as the FWHM peak width in hertz, as calculated from the most prominent peak in the fitted basis spectrum. If the MCMC algorithm is used, the quality control metrics are calculated over all samples.

#### 2.2.4 Reporting and display

The FSL‐MRS modules generate self‐contained interactive HTML reports (Plotly, Montréal, Canada), which can be viewed and interacted with in the user’s web browser. All components of the processing module (Table 1) produce short HTML reports that can be combined into a single interactive report for each instance of data using the packaged *merge_mrs_reports*.

**FIGURE 2:**
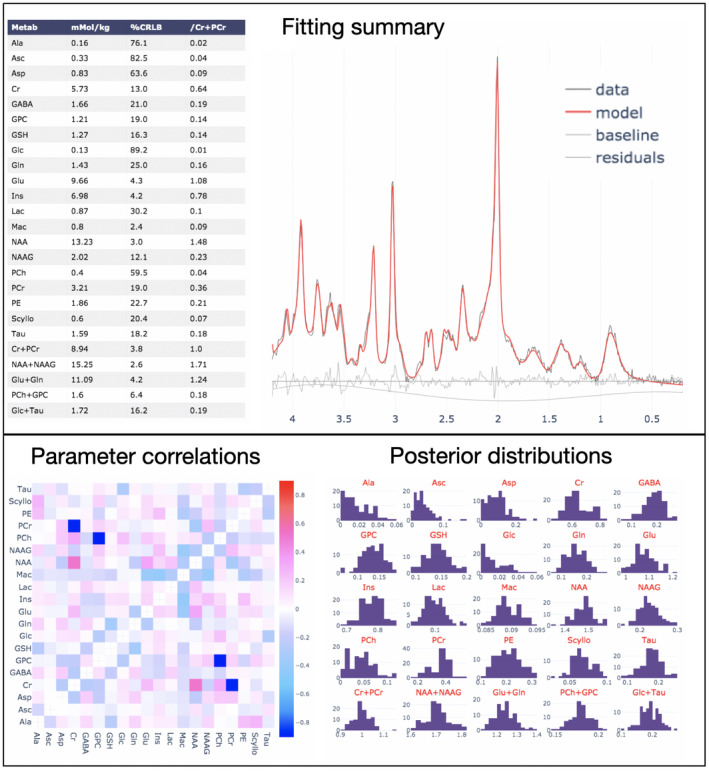
Extracts of the interactive HTML fitting report. Top: Metabolite concentrations summary and fit overlaid on data. Individual plots can be toggled on and off interactively. Bottom: Correlations between metabolite concentrations from the Markov chain Monte Carlo (MCMC) sampling and marginal posterior distributions of the metabolite concentrations. A full interactive fitting and preprocessing report is included as Supporting Information. Abbreviations: Ala, alanine; Asc, ascorbate; Asp, aspartate; Cr, creatine; GABA, γ‐aminobutyric acid; Glc, glucose; Gln, glutamine; Glu, glutamate; GPC, glycerophosphocholine; GSH, glutathione; Ins, myo‐inositol; Lac, lactose; Mac, macromolecules; NAA, N‐acetyl aspartate; NAAG, N‐acetyl aspartate glutamate; PCh, phosphocholine; PCr, phosphocreatine; PE, phosphorylethanolamine; Scylio, scyllo‐inositol; Tau, taurine

An interactive report is generated for each fit displaying the fitted spectrum, model fit, residuals and concentrations (Figure 1), concentration posterior distributions, metabolite covariances, and scaled basis spectra (Figure 2). The report also contains summaries of SNR and linewidth quality‐control parameters for each fitted metabolite. The user, therefore, can quickly assess the quality of SVS data and fit visually in one location. Results of the fitting and quality‐control metrics are also available as comma‐separated value files from the command‐line programs and as Pandas objects in memory.^34^

**FIGURE 3:**
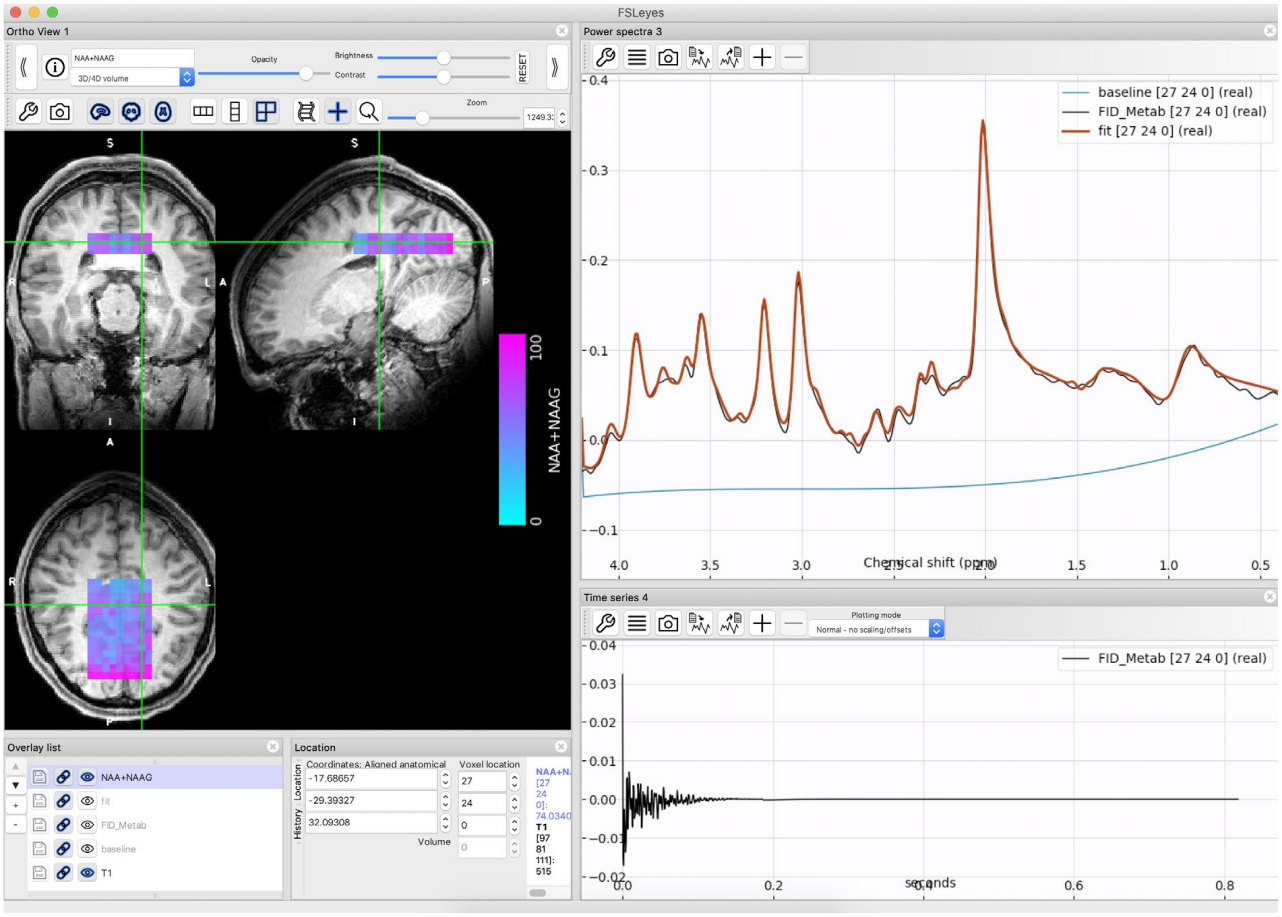
Magnetic resonance spectroscopy imaging in FSLeyes. The results of processing and fitting of MRSI data are stored in 4D NIFTI files and can be viewed in a suitable viewer such as FSLeyes. Here, a map of total NAA +NAAG as measured using CONCEPT (concentric circle echo‐planar trajectories; data set 3) is overlaid on a T_1_‐weighted image. In the lower panel, the real part of the time‐series data for the selected voxel is seen on the left, and on the right the real part of the spectral data is overlaid with the FSL‐MRS fit and baseline estimate

Visualization of both time and frequency‐domain MRSI data alongside structural imaging data can be achieved using the FSL package tool FSLeyes^35^ (Figure 3).

### 2.3 Interactive FSL‐MRS

In addition to the command‐line tools described previously, FSL‐MRS may be run in an “interactive” way by loading the underlying python libraries into an interactive IPython environment. The same functionality and reporting interfaces that are available on the command line are also available interactively. In this way, FSL‐MRS allows prototyping of new processing pipelines and tools, while also providing familiarity for users of interactive scripting languages.

### 2.4 Bayesian fitting

The FSL‐MRS toolbox implements linear combination modeling for fitting of basis spectra to data using Bayesian statistics, to find an optimal solution. This method of fitting is robust, while also outputting full posterior distributions of fitted metabolite concentrations to estimate concentration covariances and uncertainties.

The fitting module contains a standard fitting model appropriate for the fitting of a single, independent spectrum. However, the fitting framework can accept an arbitrary forward model.

#### 2.4.1 Standard fitting model

The model for the complex‐domain spectrum is

(1) $$Y\left(\nu \right)=B\left(\nu \right)+\exp\left[i\left(\phi_{0}+\nu \phi_{1}\right)\right]\sum_{g=1}^{N_{G}}\sum_{l=1}^{N_{g}}C_{l,g}M_{l,g}\left(\nu ;\gamma_{g},\sigma_{g},\varepsilon_{g}\right)$$

(2) $$M_{l,g}\left(\nu ;\gamma_{g},\varepsilon_{g}\right)=F\left\{m_{l,g}\left(t\right)\exp\left[‐\left(\left(\gamma_{g}+\sigma_{g}^{2}t\right)+i\varepsilon_{g}\right)t\right]\right\}$$

where ν denotes frequency; $B\left(\nu \right)$ describes an *N*th‐order complex polynomial estimate of the baseline; the second term applies a global zeroth and first‐order phase; and the final term is the sum of all scaled, shifted, and broadened metabolite basis spectra $M_{l,g}\left(\nu ;\;\gamma_{g},\sigma_{g},\varepsilon_{g}\right)$. To avoid overfitting, there is no flexibility in the metabolite line shapes beyond shifting (ε) and broadening (γ, σ), which can be flexibly applied to *N*_G_ groups of metabolites (where each metabolite belongs to one and one only group). F is the Fourier transform, and m_l,g_(*t*) is the inverse Fourier transform of $M_{l,g}\left(\nu ;\;0,0,0\right)$.

No prior information or constraints on relative metabolite scaling is incorporated. The default polynomial baseline is second‐order, but can be specified (or disabled entirely) by the user.

#### 2.4.2 Optimization

Initialization is achieved using the truncated Newton algorithm as implemented in the SciPy package.^36^, ^37^ The final fit is carried out over all model parameters using Metropolis‐Hastings (an MCMC algorithm).^18^ The truncated Newton initialization can be used independently of the subsequent MCMC fit to provide a fast point estimate of the metabolite concentration. In this work and in the summary reports generated by FSL‐MRS, point estimates of the metabolite concentrations from the MCMC algorithm are the arithmetic mean of the posterior distribution.

The forward model in Equation 1 is combined with an additive Gaussian white noise to produce the Likelihood function (which combines both real and imaginary parts of the model prediction and data). The noise‐variance parameter is integrated out with a Jeffrey’s (1/x) prior. Priors on the concentration parameters are set to broad zero‐mean half‐Gaussians (ie, with positivity constraint). Each of the line‐broadening parameters (γ, σ) are set to broad Gaussians (SD of 2.5 Hz) with a small, positive center (5 Hz) and positivity constraints. Thus, the prior is centered at an additional 10 Hz of line broadening in addition to the linewidth of the basis spectra. Shift and phase priors are set to broad Gaussians centered at zero, with no additional constraints. Priors can be disabled (set to uniform) by the user. The baseline parameters are estimated in the initial nonlinear fitting, then kept fixed in the MCMC stage. More details of the optimization choices (including initialization, priors, and likelihood model) can be found in the “Optimization details” section of the Supporting Information. Note that these details pertain to the results shown in this paper. The online software documentation will be kept up‐to‐date should these optimization decisions change in future releases.

#### 2.4.3 Treatment of macromolecular signals

Macromolecular signal is observed as broad resonances in short TE spectra. The signal arises from amino acid residues of peptides. The methods described in section 2.2.2 are not suitable for the creation of macromolecular basis spectra: Macromolecular resonances are broad distributions of chemical shifts arising from many different peptide molecules rather than a single metabolite molecule. The FSL‐MRS toolbox, therefore, uses empirically measured macromolecular signal (eg, from metabolite T_1_‐nulled acquisitions) as a basis. The complex polynomial‐based baseline model is not designed to describe macromolecular signals.

For situations in which empirically measured macromolecular signals are not available, simulated basis spectra, generated at known chemical shift positions, may be added to the set of basis spectra automatically. The details of these basis spectra at the time of writing are listed in Supporting Information Table S5) and in the online documentation. Users may add additional peaks or modify the defaults. In all cases, macromolecular basis spectra are treated identically to metabolite basis spectra, but are grouped separately to allow suitable separate optimization of frequency shift and line‐broadening parameters.

### 2.5 Validation of fitting

All methods in this work refer to version 1.0.5 of FSL‐MRS.

#### 2.5.1 Simulation

Independently created simulated data were used to validate FSL‐MRS. The simulated data were created by Malgorzata Marjanska, Dinesh Deelchand, and Roland Kreis for the ISMRM MRS Study Group’s Fitting Challenge.^38^ The data consists of 21 data sets (without artifacts) with varying SNR, linewidths, line shapes, metabolite concentrations, and macromolecule content. Briefly, data sets 0‐2 have increasing widths of Lorentzian line shapes; 3‐5 have increasing widths of Gaussian line shapes; 6‐9 vary the concentration of γ‐aminobutyric acid/glutathione; 10 have no macromolecular content; 11‐13, 14‐16, 17‐19, and 20 have different spectral SNRs (20, 30, 40, and 160, respectively). The data simulate a 3T point‐resolved spectroscopy sequence with a T_E_ of 30 ms.

Both water‐suppressed and unsuppressed data are provided in an already preprocessed state. Basis spectra for 17 metabolites, including a macromolecular baseline, were provided by the challenge authors. The metabolites included are alanine, ascorbate, aspartate, γ‐aminobutyric acid, glucose, glutamine (Gln), glutamate (Glu), glutathione, glycine, myo‐inositol (Ins), lactate (Lac), N‐acetyl aspartate (NAA), N‐acetyl aspartate glutamate (NAAG), phosphorylethanolamine, scyllo‐inositol, and taurine. In the analysis, the following metabolites were treated together: NAA + NAAG, Glu + glutamine, glycerophosphocholine (GPC) + phosphocholine (PCh), creatine (Cr) + phosphocreatine (PCr), glucose + taurine, and myo‐inositol + glycine. True concentration values for each metabolite in each data set were supplied by the Fitting Challenge authors in a private communication.

Fitting was assessed for both the Newton and MCMC algorithms. The polynomial baseline was restricted to zeroth order, and fitting was carried out between 0.2 and 4.2 ppm. After fitting, scaling of the raw metabolite concentrations was carried out using the unsuppressed water data, and concentration values were scaled, accounting for provided tissue‐volume fractions.^32^

Fitting performance was assessed using the mean and median percentage difference and absolute concentration difference from the true concentration values for each metabolite in all data sets. In addition, a summary statistic for each metabolite in each spectrum was calculated from the MCMC estimated posterior distribution as follows:

(3) $$\frac{\mu_{\left[M\right]}‐{\left[M\right]}_{\text{True}}}{\sigma_{\left[M\right]}},$$

where $\mu_{\left[M\right]},\sigma_{\left[M\right]}$ are the mean and SD of the fitted concentration of metabolite M, and [M]_True_ is its true value. Intuitively, this statistic can be interpreted as “how many SDs away from the true value is our estimate?”

#### 2.5.2 Phantom

Validation of unsuppressed, water‐scaled concentrations was carried out in a uniform aqueous phantom (SPECTRE; Gold Standard Phantoms, London, United Kingdom) containing six metabolites (N‐acetyl aspartate, Cr, Cho, Ins, Glu, and Lac) using a previously published stimulated echo acquisition mode (STEAM) sequence at 7 T.^39^, ^40^ The sequence parameters were 11‐ms TE, 32‐ms mixing time (T_M_), 10‐second TR, 4096 samples, and 6000‐Hz bandwidth. Basis spectra were created using FSL‐MRS. Basis spectra simulation used fully described (nonideal) pulse shapes, gradients, and timing parameters, and were conducted using a spatial resolution of 30 points in each gradient dimension. The concentrations of six metabolites was determined from 5‐Hz exponentially line‐broadened spectra from the phantom. This broadening was introduced to permit the use of the standard in vivo Bayesian priors in the optimization. An additional doublet near 1.4 ppm was observed in the spectrum. It was established to be contaminant of the Lac feedstock used to create the phantom. It was fitted as alanine and included in the Lac concentration. Absolute concentrations were calculated by referencing the integral of the scaled creatine spectrum to an unsuppressed water spectrum taken to be equivalent to 55.5 M H_2_O. The T_2_s were estimated from water, and an average of metabolite singlet linewidths and concentrations were scaled for metabolite and water T_2_ relaxation.

#### 2.5.3 In vivo

The FSL‐MRS fitting was validated against LCModel (version 6.3‐1M)^13^ in three in vivo data sets. The data sets covered different brain regions, sequences and field strengths, and are summarized in Table 2. Data sets 1 and 2 contain SVS data using STEAM and SPECIAL (special inversion at lipid)^41^ sequences, respectively, and data set 3 contains 2D multivoxel MRSI data collected using density‐weighted CONCEPT (concentric circle echo‐planar trajectories) with a semi‐LASER volume‐selection module.^2^ The STEAM and SPECIAL data were processed using *fsl_mrs_proc*.

**TABLE 2:** Description of in vivo data sets used for validation

No.	Sequence	B_0_ (T)	Subjects	Voxels (brain regions)	Measured MM?	Vendor	References
1	STEAM	7	37	3 (ACC, OCC, putamen)	Yes	Siemens	Ref. 40
2	SPECIAL	3	220	1 (PCC)	Yes	Siemens	Ref. 42
3	CONCEPT	3	8	126 (calcarine sulcus)	No	Siemens	Ref. 43

Abbreviations: ACC, anterior cingulate cortex; CONCEPT, concentric circle echo‐planar trajectories; OCC, occipital cortex; PCC, posterior cingulate cortex; SPECIAL, special inversion at lipid; STEAM, stimulated echo acquisition mode.

All subjects in these data sets were recruited in a manner approved by the appropriate research ethics committee for each originating study (see references in Table 2).

Identical basis spectra were used in both FSL‐MRS and LCModel. Basis spectra for data sets 1 and 2 were created in FSL‐MRS using fully described RF pulses and gradients, coherence filtering, and were simulated with 30 spatial points in each gradient dimension. The basis spectra consisted of 19 and 17 simulated metabolites, respectively, to match previous analyses. Previously measured macromolecular spectra from metabolite inversion‐nulled sequences were included in the basis spectra. For data set 3, existing basis spectra (as described in Steel et al^43^) were used. They were simulated in the simulation module of VeSPA (Versatile Simulation, Pulses and Analysis)^44^ and consist of 19 simulated metabolites. Macromolecular spectra were not included; instead, eight LCModel or FSL‐MRS‐simulated Gaussian macromolecule resonances were included in the analysis at the following positions: 0.91, 1.21, 1.43, 1.67, 1.95, 2.08, 2.25, and 3.00 ppm. For all data sets, default “concentration ratio priors” (also referred to as soft constraints) for metabolites were specified for the LCModel fit (ie, the LCModel control file parameter “NRATIO” was set to the default value of 12, corresponding to the first 12 ratio priors specified in §11.8 of the LCModel manual, of which numbers 8‐12 are active in data sets 1 and 2, and numbers 1‐6 and 8‐12 are active in data set 3). In LCModel, the baseline flexibility parameter DKNTMN was set to 0.25, slightly above the default (0.15), while in FSL‐MRS, the baseline order was set to second order, second order, and fourth order for data sets 1, 2, and 3, respectively.

Data sets 1 and 2 were fitted using LCModel and FSL‐MRS (MCMC algorithm). Data set 3 was fit in LCModel and FSL‐MRS (Newton algorithm) for speed. Highly correlated peaks (correlation coefficient < −0.5) were combined (eg, Cr + PCr, NAA + NAAG, PCh + GPC, Glu + Gln). Metabolite concentrations were expressed as a ratio to total creatine (Cr + PCr) and as molality concentrations using unsuppressed water as an internal reference. T_2_ relaxation was accounted for, but the unsuppressed water peak was assumed to correspond to pure water, as anatomical images for tissue segmentation were not available for all data sets.

Data were compared voxel‐wise for each metabolite in each data set using the Pearson correlation coefficient and Bland‐Altman bias and limits of agreement.^45^ Bias and limits of agreement were calculated in concentration units or as ratios to total creatine (Cr + PCr). They were summarized by expressing the bias as a percentage of the mean concentration value, and the limits of agreement as the width of the 95% confidence intervals expressed as a percentage of the mean value before averaging these values across all metabolites. In the comparisons, data were excluded if the estimated percentage Cramér–Rao lower bounds on the metabolite concentrations exceeded 100% for either FSL‐MRS or LCModel, or if the fitted value was more than four SDs from the mean value for that metabolite in that data set. Metrics were calculated for both the water‐scaled concentrations (water) and metabolite ratios (total creatine) for all metabolites, excluding the combined values (all), and for all metabolites including combined values but excluding those that were combined (combined).

## 3 RESULTS

### 3.1 Output and reports

Figure 2 shows extracts of an example FSL‐MRS fitting report. The extracts include a summary of the fit and metabolite concentrations, MCMC‐estimated correlations between metabolite concentrations, and visualizations of the MCMC estimated distributions of the metabolite concentrations. Example fully interactive HTML reports for both fitting and processing are included as Supporting Information. The same reports can be generated from example data included in the FSL‐MRS package.

Figure 3 shows the results of fitting an MRSI grid of voxels from a single‐density‐weighted CONCEPT from data set 3 (Table 2). The NIfTI format viewers such as FSLeyes can be used to simultaneously view anatomical images, fitted metabolite concentrations, the spectral data, and the FSL‐MRS fit. In Figure 3, the total NAA concentrations are overlaid on a T_1_‐weighted image centered around the calcarine sulcus.

Fitting results may be exported in NIfTI or comma‐separated value format, or carried forward in Python for further analysis.

### 3.2 Validation

#### 3.2.1 Simulation

**FIGURE 4:**
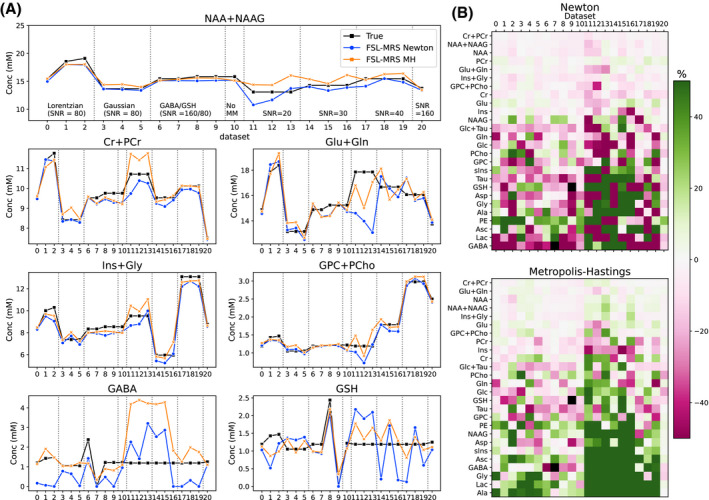
Simulation validation. A, Comparison of FSL‐MRS‐measured concentrations for each MRS Fitting Challenge data set for seven metabolites. B, Percentage difference from true values for all metabolites for all data sets. The metabolites are sorted by mean difference. Both fitting algorithms (Newton [top] and Metropolis Hastings [bottom]) are shown

Figure 4 summarizes the results of the validation on simulated data for all metabolites in all simulated data sets. Detailed plots for each metabolite are included as Supporting Information Figure S1.

For all metabolites across all 21 data sets, the Newton algorithm achieved a mean (median) absolute concentration difference of 0.60 (0.41) mM and a mean (median) absolute percentage difference of 30.6% (14.9%). The MCMC algorithm achieved a mean (median) absolute concentration difference of 0.60 (0.37) mM and a mean (median) absolute percentage difference of 35.2% (11.9%). For the five most prominent signals (NAA + NAAG, Cr + PCr, Glu + Gln, Ins + Gly, and GPC + PCh), the MCMC algorithm had a mean difference of 0.48 mM or 5.4%. The mean (± SD) number of SDs from the true value (Equation 3) was 0.57 ± 0.43. A total of 98.9% of true metabolite concentration values were between the 5th and 95th percentiles of the MCMC‐estimated posterior distributions. Uncombined choline (GPC and PCh) and creatine (PCr and Cr) peaks were excluded from the calculation, as described in the original Fitting Challenge results.

#### 3.2.2 Phantom

**FIGURE 5:**
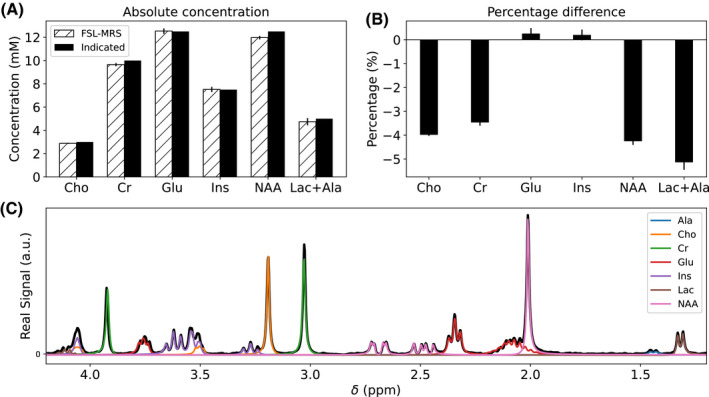
Phantom validation. A, Absolute concentration of fitted metabolite compared with known concentrations. Cramér–Rao lower bound is indicated by vertical bars. B, Percentage difference from true value. C, Data overlaid with FSL‐MRS fit (each metabolite fit is shown in a different color). The doublet at 1.4 ppm is fitted as Ala and included with Lac

Figure 5 summarizes the results of the absolute concentration validation in phantom. The mean absolute percentage difference from the true concentration across all metabolites was 3.39% (range ‐7.1% [Lac] to 1.1% [Glu]).

The single creatine basis set used was unable to simultaneously fit both creatine singlet peaks (CH_2_ at 3.93 ppm, CH_3_ at 3.03 ppm) with small residuals. The creatine singlets have been observed to have different T_2_ relaxation properties, which is unmodeled in these basis spectra,^46^ and could account for the observed difference in fit quality between peaks.

#### 3.2.3 In vivo

Table 3 summarizes the in vivo fitting validation correlations and Bland‐Altman metrics for each data set. The per‐metabolite correlations for each of the three data sets and for both referencing methods are provided in Supporting Information Table S6.

**TABLE 3:** Summary of in vivo validation—correlation and Bland‐Altman statistics

Data set	Scaling	Correlation *all*	Correlation *combined*	% Bias *all*	% Bias *combined*	% LoA *all*	% LoA *combined*
1) 7T STEAM	Water	0.69 ± 0.18	0.74 ± 0.19	22 ± 21	14 ± 12	120 ± 71	93 ± 64
	tCr	0.71 ± 0.17	0.75 ± 0.18	20 ± 21	11 ± 11	108 ± 63	84 ± 64
2) 3T SPECIAL	Water	0.68 ± 0.13	0.75 ± 0.12	44 ± 25	45 ± 27	414 ± 329	369 ± 345
	tCr	0.77 ± 0.18	0.81 ± 0.14	34 ± 34	28 ± 33	277 ± 337	259 ± 408
3) 3T CONCEPT	Water	0.53 ± 0.16	0.56 ± 0.14	37 ± 22	37 ± 20	216 ± 109	193 ± 92
	tCr	0.54 ± 0.15	0.58 ± 0.13	27 ± 22	24 ± 23	162 ± 90	138 ± 96

Note

All values are presented as mean ± SD.

Abbreviations: All, all metabolites (excluding combined); combined, after combination (excludes those combined); LoA = limits of agreement (width of 95% confidence interval); tCr, total creatine.

**FIGURE 6:**
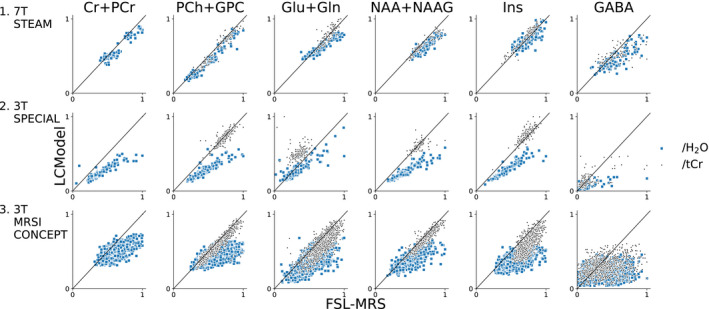
Summary of in vivo validation. Correlation plots of a selected group of metabolites for each validation data set. Solely for display purposes, ratios to unsuppressed water and total creatine (Cr + PCr) are normalized to the maximum value fitted by either FSL‐MRS or LCModel. Correlation plots for all metabolites are shown in the Supporting Information

Mean correlations between FSL‐MRS and LCModel in all data sets achieved a correlation over 0.5, and correlations were similar for both water‐scaled concentrations and metabolite ratios. Correlations for the combined metabolite group were higher than the uncombined “all” group, as the high SNR combined metabolites (Cr + PCr, PCh + GPC, Glu + Gln, NAA + NAAG, and Ins) achieved correlations in the range of 0.81‐0.98 for all data sets. The highest metabolite correlation across all three data sets was achieved by total choline (0.85), the lowest was glucose (0.34), and the median per‐metabolite correlation was 0.70. Figure 6 shows scatter plots for a sample of metabolites for each data set (for display purposes only, concentrations and ratios were normalized to the maximum value fitted by either LCModel or FSL‐MRS). The scatter plots for all metabolites for each data set are included in Supporting Information Figures S2‐S4).

When averaged across all metabolites, Bland‐Altman metrics showed a consistent bias for higher metabolite concentrations (mean of 14%‐37%) and metabolite ratios (mean of 11%‐24%) in FSL‐MRS compared with LCModel. Bias was higher for water‐scaled concentrations compared with metabolite ratios, and lower for the combined metabolites. Bland‐Altman plots are shown for high‐SNR metabolites (Cr + PCr, PCh + GPC, Glu + Gln, NAA + NAAG, and Ins) in Supporting Information Figure S5. These metabolites showed much lower bias when referenced to total creatine (Supporting Information Table S7) versus water referencing (Supporting Information Table S8).

## 4 DISCUSSION

The FSL‐MRS toolbox is an end‐to‐end spectroscopy analysis package. It is designed to be used flexibly: either implementing all stages of the MRS analysis pipeline or being used as a modular part of another pipeline. The package is scriptable on the command line, requiring no interaction, and is suitable for analysis of large data sets and for deployment with high‐performance computing, or it can be used interactively, such as for pipeline prototyping and novel analyses.

The FSL‐MRS package achieves modularity by operating on data stored in a standard file type, NIfTI, which is already in use throughout neuroimaging. Existing packages for handling NIfTI data exist in many programming languages (eg, NiBabel in Python and the “image processing toolbox” in *MATLAB* [The MathWorks, Natick, MA]), enabling FSL‐MRS to be integrated with other MRS analysis programs. Results generated in NIfTI format allow straightforward integration of MRS data into multicontrast analysis in existing neuroimaging toolboxes (eg, FSL). Both FSL‐MRS and Python are open source and free for academic and noncommercial use.

The package includes visualization modules for generating interactive HTML reports, viewable in a wide range of internet browsers. Visualization of data and fitting results can also be accomplished in NIfTI format viewers due to the use of standard data types. Visualization of data remains important, while fully automatic quality control of MRS data remains not widespread.^20^

Validation of the FSL‐MRS fitting module was carried out on simulation, phantom, and in vivo data. Validation on the simulated data showed low absolute concentration errors except in those data sets with low spectral SNRs (20 and 30) and in peaks with low SNR and high correlation with neighbors (eg, GABA). MCMC fitting of metabolites with low concentrations generates skewed distributions that are not well described with a single point statistic (the mean value), which may contribute to the marginally better performance of the Newton algorithm. Phantom validation indicated that the package correctly implements calculation of absolute concentrations using scaling to unsuppressed water in the case of pure water.

In in vivo data, the validation was against LCModel, an established and widely used fitting program. Bias toward higher metabolite ratios in FSL‐MRS was observed for water‐scaled concentrations and, to a lesser extent, for relative metabolite ratios. The latter might arise from FSL‐MRS not implementing priors between relative metabolite concentrations, a default setting in LCModel that was enabled in this analysis. Soft constraints in LCModel restrict certain metabolite concentration ratios (ratios of low‐SNR metabolites to a weighted average of NAA, total creatine, and total choline) to be within a certain normally distributed range. The larger differences in water‐scaled metabolite concentrations are likely due to the different implementations of flexible baselines in the two packages. LCModel and other programs^47^, ^48^ implement a spline‐based baseline; in this work we chose a complex polynomial implementation. The inclusion of a zeroth‐order term allows a uniform vertical shift in baseline across all frequencies, not typically possible with spline baselines. Across all voxels in the MRSI data set, negative correlations were observed between absolute concentrations and the zeroth‐order polynomial baseline parameters. A baseline below zero will increase the reference peak’s absolute integral and result in a large ratio when compared with the integral of unsuppressed water. The effect of the precise implementation of flexible baselines on metabolite concentrations in fitting packages is complex,^49^, ^50^ with dependence on acquisition, description of macromolecules in basis spectra, and optimization algorithm. The FSL‐MRS’s implementation of a complex polynomial baseline does not offer a solution to this complexity, but the implementation is simple to understand and implement, is unlikely to cause overfitting, and is only parametrized by 2n nuisance parameters for an order‐n baseline. If enabled, the MCMC algorithm enables the user to calculate the covariance of the baseline parameters with the metabolite concentrations. An example MCMC correlation matrix of a single spectrum from data set 1 (7T STEAM), including baseline parameters, shows that baseline parameters only correlate strongly with the macromolecule concentration (Supporting Information Figure S6). Efforts to widely measure and account for differences in fitting software^51^ will be essential to provide program quality assurance and allow for meaningful use of pooled data analyzed using different tools.

Fitting using the MCMC algorithm allows the user to generate the full posterior distribution for each fitted parameter, including metabolite concentrations. This information is essential to understanding the uncertainties inherent in the estimation of the parameters. It also offers the opportunity to carry forward this information into subsequent study analysis, reducing the need for arbitrary quality cutoffs to be used. However, fitting using the MCMC algorithm is inherently slower than methods that provide only point estimates, taking tens of seconds rather than seconds to compute the results for each voxel. It may be possible to achieve the estimation of the posterior distributions in the time frame of a few seconds using a variational inference optimizer, which is under development.^52^

Operation of the package still requires the user to provide expert knowledge in two places: data conversion and generation of basis spectra. At the data‐conversion stage, the user must either use a file format understood by *spec2nii* and must interpret the structure of the data within that format, or provide a full conversion, including orientation information, for their own data format. Generating correct basis spectra requires the user to provide an accurate description of the RF pulses, timings, and gradients in the localization module of their sequence. Documentation for the package has been created to mitigate difficulties in these stages. The *fsl_mrs* SVS fitting can interpret a select few other formats (LCModel “.RAW” and jMRUI “.txt”).

The FSL‐MRS MCMC fitting module accepts an arbitrary forward model. In future work we intend to use this framework to investigate the advantages of fitting multiple spectra simultaneously with a specialist model (such as for diffusion‐weighted, edited, or functional MRS). The FSL‐MRS package is under continued development and refinement; online documentation provides the latest and up‐to‐date information on the package. Currently, the package is optimized for 3T and 7T in vivo human 1H‐MRS data, fitting routines, basis spectra, and prior knowledge, which need to be suitably modified for a greater range of data.

## 5 CONCLUSIONS

We have presented a new end‐to‐end spectroscopy processing package that incorporates Bayesian fitting of spectra. The package is open‐source, modular, and freely available. This work has provided validation of the package by simulation, in phantom, and in three in vivo data sets. The complete package is available for download at git.fmrib.ox.ac.uk/fsl/fsl_mrs, through the open‐source package management system Conda (Continuum Analytics, Austin, TX), and will be available as part of FSL (fsl.fmrib.ox.ac.uk).

## Supporting information


**FIGURE S1** Simulation validation results for all metabolites
**FIGURE S2** All metabolite correlations for data set 1 (STEAM [stimulated echo acquisition mode], 7 T)
**FIGURE S3** All metabolite correlations for data set 2 (SPECIAL [special sequence at lipid], 3 T)
**FIGURE S4** All metabolite correlations for data set 3 (MRSI, 3 T)
**FIGURE S5** Bland‐Altman plots for selected metabolites
**FIGURE S6** Markov chain Monte Carlo (MCMC) parameter correlations of a single data set
**TABLE S1** File formats supported by spec2nii
**TABLE S2** Quantification constants: tissue–water density
**TABLE S3** Quantification constants: T_1_ values
**TABLE S4** Quantification constants: T_2_ values
**TABLE S5** Synthetic macromolecular basis spectra specification
**TABLE S6** In vivo validation per‐metabolite Pearson correlations
**TABLE S7** Bland‐Altman statistics for creatine‐referenced high‐SNR metabolite peaks
**TABLE S8** Bland‐Altman statistics for water‐referenced high‐SNR metabolite peaks
